# Cross-Linking in Collagen by Nonenzymatic Glycation Increases the Matrix Stiffness in Rabbit Achilles Tendon

**DOI:** 10.1080/15438600490277860

**Published:** 2004

**Authors:** G. Kesava Reddy

**Affiliations:** 1 Department of Physical Therapy and Rehabilitation Sciences University of Kansas Medical Center Kansas City Kansas USA; 2 Baylor-Sammons Cancer Center Suite 185 3535Worth Street Dallas TX 75246 USA

## Abstract

Nonenzymatic glycation of connective tissue matrix proteins is a major contributor to the pathology of diabetes and aging. Previously the author and colleagues have shown that nonenzymatic glycation significantly enhances the matrix stability in the Achilles tendon (Reddy et al., 2002, *Arch. Biochem. Biophys.*, 399, 174–180). The present study was designed to gain further insight into glycation-induced collagen cross-linking and its relationship to matrix stiffness in the rabbit Achilles tendon. The glycation process was initiated by incubating the Achilles tendons (n = 6) in phosphate-buffered saline containing ribose, whereas control tendons (n = 6) were incubated in phosphate-buffered saline without ribose. Eight weeks following glycation, the biomechanical attributes as well as the degree of collagen cross-linking were determined to examine the potential associations between matrix stiffness and molecular properties of collagen. Compared to nonglycated tendons, the glycated tendons showed increased maximum load, stress, strain, Young's modulus of elasticity, and toughness indicating that glycation increases the matrix stiffness in the tendons. Glycation of tendons resulted in a considerable decrease in soluble collagen content and a significant increase in insoluble collagen and pentosidine. Analysis of potential associations between the matrix stiffness and degree of collagen cross-linking showed that both insoluble collagen and pentosidine exhibited a significant positive correlation with the maximum load, stress, and strain, Young's modulus of elasticity, and toughness (*r* values ranging from .61 to .94) in the Achilles tendons. However, the soluble collagen content present in neutral salt buffer, acetate buffer, and acetate buffer containing pepsin showed an inverse relation with the various biomechanical attributes tested (*r* values ranging from .22 to .84) in the Achilles tendons. The results of the study demonstrate that glycation-induced collagen cross-linking is directly associated with the increased matrix stiffness and other mechanical attributes of the tendon.


*Experimental Diab. Res.*, 5:143–153, 2004
ISSN: 1543-8600 print / 1543-8619 online
DOI: 10.1080/15438600490277860

Taylor & Francis
Taylor & Francis Group

### Cross-Linking in Collagen by Nonenzymatic Glycation Increases the Matrix Stiffness in Rabbit Achilles Tendon

**G. Kesava Reddy**

*Department of Physical Therapy and Rehabilitation Sciences, University of Kansas Medical Center, Kansas City, Kansas, USA*

**Nonenzymatic glycation of connective tissue matrix proteins is a major contributor to the pathology of diabetes and aging. Previously the author and colleagues have shown that nonenzymatic glycation significantly enhances the matrix stability in the Achilles tendon (Reddy et al., 2002, *Arch. Biochem. Biophys.*, 399, 174–180). The present study was designed to gain further insight into glycation-induced collagen cross-linking and its relationship to matrix stiffness in the rabbit Achilles tendon. The glycation process was initiated by incubating the Achilles tendons (n = 6) in phosphate-buffered saline containing ribose, whereas control tendons (n = 6) were incubated in phosphate-buffered saline without ribose. Eight weeks following glycation, the biomechanical attributes as well as the degree of collagen cross-linking were determined to examine the potential associations between matrix stiffness and molecular properties of collagen. Compared to nonglycated tendons, the glycated tendons showed increased maximum load, stress, strain, Young's modulus of elasticity, and toughness indicating that glycation increases the matrix stiffness in the tendons. Glycation of tendons resulted in a considerable decrease in soluble collagen content and a significant increase in insoluble collagen and pentosidine. Analysis of potential associations between the matrix stiffness and degree of collagen cross-linking showed that both insoluble collagen and pentosidine exhibited a significant positive correlation with the maximum load, stress, and strain, Young's modulus of elasticity, and toughness (*r* values ranging from .61 to .94) in the Achilles tendons. However, the soluble collagen content present in neutral salt buffer, acetate buffer, and acetate buffer containing pepsin showed an inverse relation with the various biomechanical attributes tested (*r* values ranging from .22 to .84) in the Achilles tendons. The results of the study demonstrate that glycation-induced collagen cross-linking is directly associated with the increased matrix stiffness and other mechanical attributes of the tendon.**

**Keywords** Collagen Cross-Linking; Connective Tissue Matrix; Glycation; Maillard Reaction; Stiffness; Tissue Biomechanics

Nonenzymatic glycation, also known as the Maillard reaction between reducing sugars and proteins, contributes to the chemical aging of tissue proteins in vivo and to the accelerated aging of proteins in diabetes mellitus [1–3]. In this reaction, sugars react reversibly with the free amino group of proteins to form unstable Schiff bases, which then undergo an intramolecular rearrangement to form a stable Amadori product [4, 5]. These Amadori products are believed to undergo a series of reactions to form heterogeneous complex fluorophores and chromophores collectively referred to as advanced Maillard products or advanced glycation end products (AGEs). The production of these AGE products have been implicated in the etiology of the long-term complications of several human afflictions, such as diabetes [1, 3, 6], aging [7], atherosclerosis [8], fibromyalgia [9], uremia [10, 11], Alzheimer's disease [12, 13], and renal failure [14].

Collagen provides many basic functional attributes of the connective tissues in the body. Glycation can affect collagen in a number of ways: its ability to form precise supramolecular aggregates, the alterations in its charge profile, defects in the

Received 29 June 2003; accepted 11 September 2003.
These studies were supported by a grant from the American Heart Association, the Heartland Affiliate Research Program (AHA-9960226Z), to GKR.
Address correspondence to Dr. G. Kesava Reddy, Baylor-Sammons Cancer Center, Suite 185, 3535 Worth Street, Dallas, TX 75246, USA. E-mail: kreddy@yahoo.com formation of its fibrils, and hence its interaction with cells. In addition to the occurrence of biochemical and morphological manifestations, glycation has been shown to alter the biomechanical functioning of the connective tissues [15–21]. Both in vivo and in vitro studies have shown that reducing sugars, such as glucose and ribose, react with the amino groups of collagen and other proteins and form cross-linked AGEs in the tissue. These AGEs accumulate over time and lead to the functional impairment of the tissue. Earlier studies have shown that glycation alters the structural properties of collagen [22]. These earlier studies investigated the effect of glycation on either the biochemical nature of collagen or the biomechanical functions of the tissue. However, the interrelationship between the altered biochemical properties of collagen and the altered biomechanical functions of the tendon produced by glycation requires further investigation.

Despite recent advances in our understanding of glycation of proteins, there is relatively little evidence available concerning the glycation-induced collagen cross-linking that is directly responsible for the altered stiffness of the tissue. Recently, we have shown that nonenzymatic glycation alters both biomechanical and biochemical functioning of the connective tissue matrix in rabbit Achilles tendon [23]. In the present study, we report a direct analysis of the potential associations between the degree of collagen cross-linking and matrix stiffness in Achilles tendon following nonenzymatic glycation.

### MATERIALS AND METHODS

Achilles tendons from white male New Zealand rabbits, aged 12 to 16 weeks, were collected and stored at −70°C. The procedures for tendon excision were described in detail previously [23–26].

### Glycation of Achilles Tendons With Ribose

The details of the glycation of Achilles tendons with ribose were described previously [23]. Briefly, at the start of the experiment, the tendons were thawed at room temperature and washed extensively in 20 mM phosphate-buffered saline (PBS), pH 7.4, containing 0.1% sodium azide as preservative. The specimens were divided equally into control (nonglycated) and experimental (glycated) groups ($n = 6$ per group) by random selection. The glycation process was initiated by incubating the tissue specimens with 0.2 M ribose in PBS, pH 7.4, containing 0.1% sodium azide. The tendons in the control group were incubated in PBS, pH 7.4, containing 0.1% sodium azide without ribose. The nonenzymatic glycation reaction process was allowed to proceed for 8 weeks at 29°C ± 1°C in a temperature-controlled incubator. During the initial 2 weeks, the incubation medium was changed for all samples every 3rd day. Subsequently the medium was changed every 4th day.

### Biomechanical Analysis

The analysis of the biomechanical properties of tendons was carried out using the Instron Materials Testing Device, Model 8511 (Instron, Canton, MA) as described extensively in our previous work [23, 24, 26]. Initially, the tendon specimens were secured between the clamps and pulled to rupture at a crosshead speed of 250 mm/min. The data were digitized, displayed, and a load versus displacement curve was recorded using an IBM computer. From the load/deformation curve, tensile strength, stress, strain, Young's modulus of elasticity, and toughness were calculated for each specimen.

### Biochemical Analyses

Immediately following biomechanical measurements, the ruptured Achilles tendons were collected and used for biochemical analysis. The failure site of the tendons (site of rupture) was dissected from each tendon, cut into fine pieces, and processed for biochemical analysis.

#### *Determination of the Degree of Collagen Cross-Linking*

The extent of collagen cross-linking was assessed by sequential extractions of collagen in neutral salt buffer, acetate buffer, and acetate buffer containing pepsin as described earlier

**TABLE 1**
Comparison of various biomechanical characteristics of control and glycated rabbit Achilles tendons

| Biomechanical measurements | Control Achilles tendon | Glycated Achilles tendon | Change (%) |
|---|---|---|---|
| Maximum load (N) | 292.30 ± 9.8 | 438.03 ± 20.95* | +50 |
| Maximum stress (MPa) | 7.83 ± 0.51 | 20.25 ± 2.13* | +159 |
| Maximum strain (%) | 59.59 ± 3.21 | 68.51 ± 2.93* | +15 |
| Young's modulus (MPa) | 24.89 ± 1.52 | 65.087 ± 14.41* | +161 |
| Energy absorption (mJ) | 392.30 ± 51.54 | 1038.32 ± 109.35* | +164 |
| Toughness (MPa) | 4.92 ± 0.65 | 13.58 ± 1.2563* | +176 |

*Note*. Values are mean ± SE (N = 6).
$^{*}P < .001$.

**TABLE 2**
Comparison of various biomechanical characteristics at breakpoint of control and glycated rabbit Achilles tendons

| Biomechanical measurements | Control Achilles tendon | Glycated Achilles tendon | Change (%) |
|---|---|---|---|
| Load at break (N) | 125.25 ± 9.90 | 147.66 ± 4.60* | +18 |
| Stress at break (MPa) | 3.45 ± 0.28 | 6.29 ± 0.43* | +80 |
| Strain at break (%) | 118.49 ± 7.90 | 126.96 ± 7.40 | +8 |
| Energy absorption at break (mJ) | 245.40 ± 18.60 | 280.09 ± 16.02 | +14 |

*Note*. Values are mean ± SE (N = 6).
$^{*}P < .001$.

[24, 25]. In addition, the glycation-induced cross-linking was determined using pentosidine assay as described previously [23].

*Hydroxyproline Assay*

The methods used to quantify the amount of hydroxyproline present in neutral salt soluble collagen (NSC), acid soluble collagen (ASC), pepsin soluble collagen (PSC), and insoluble collagen (ISC) have been extensively described in our previous work [23–25, 27]. Briefly, aliquots of the samples were hydrolyzed in alkali and oxidized with chloramine-T. The chromophore was developed with the addition of Ehrlich's aldehyde and the absorbance of the chromophore was measured at 550 nm. Unknown concentrations of hydroxyproline in each tissue specimen were deduced from a standard calibration curve. The total collagen content was calculated assuming that hydroxyproline comprises 14% of the total amino acids of collagen.

*Quantitation of Pentosidine*

The measurement of pentosidine levels in glycated and nonglycated tendons was determined using a previously reported procedure [23, 28], employing high-performance liquid chromatography (HPLC; Shimadzu) with a binary gradient system module managing a RF-10A spectrofluorometric detector. Briefly, both glycated and nonglycated samples were hydrolyzed in 6 N HCl for 16 hours at 110°C and freeze-dried to remove the acid. They were then reconstituted in distilled water, aliquots were taken into 0.1% trifluoroacetic acid (TFA), and analyzed by HPLC using a C18 reverse-phase column (Supelco Supelcosil 25 cm × 4.6 mm with 5-mm LC-18 pore size) equilibrated with 0.1% TFA. A gradient of 0% to 6% acetonitrile (0.1% in TFA) was run in 30 minutes at a flow rate of about 1 mL per minute. A standard curve was generated by running known quantities of pentosidine (kindly provided by Dr. Raja Khalifah) using 335 nm excitation/385 nm emission fluorescence. Elution position and the amount of fluorescence was compared to the pentosidine standard for quantitation.

*Statistical Analysis*

The results were expressed as a mean ± standard error. Statistical significances of the differences between the nonglycated and glycated Achilles tendons were evaluated using 1-way analysis of variance (ANOVA). Using Sigmastat software, a linear regression analysis was performed to correlate biochemical and biomechanical measurements. A $P$ value less than .05 was considered statistically significant.

### RESULTS

### Biomechanical Measurements

The biomechanical tests indicated significant differences in biomechanical properties between the nonglycated and glycated Achilles tendons (Tables 1 and 2). Measurements of

**TABLE 3**
Comparison of the degree of collagen cross-linking between the control and glycated rabbit Achilles tendons

| Degree of collagen cross-linking | Control Achilles tendon | Glycated Achilles tendon | Change (%) |
|---|---|---|---|
| Neutral salt soluble collagen (NSC)[a] | 4.40 ± 0.52 | 2.71 ± 0.26* | −61 |
| Acid soluble collagen (ASC)[a] | 15.61 ± 0.86 | 8.40 ± 0.52* | −48 |
| Pepsin soluble collagen (PSC)[a] | 178.60 ± 7.90 | 112.60 ± 4.7* | −29 |
| Insoluble collagen (ISC)[a] | 332.00 ± 16.10 | 426.50 ± 13.06* | +28 |
| Pentosidine[b] | 2.20 ± 0.20 | 5.90 ± 0.30* | +168 |

*Note*. Values are mean ± SE (N = 6).
[a]Expressed as $\mu$g/mg of dry tissue.
[b]Expressed as pmol/mg of collagen.
$^{*}P < .001$.

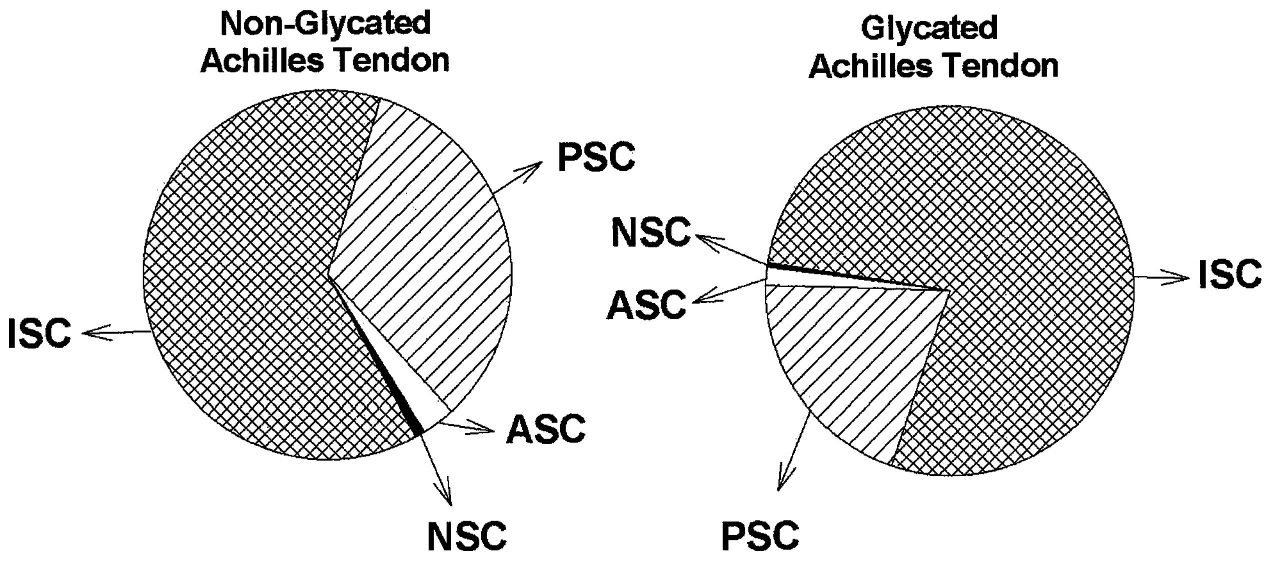

FIGURE 1
The distribution of the relative proportions of neutral salt soluble, acid soluble, pepsin soluble, and insoluble collagens in nonglycated and glycated Achilles tendons.

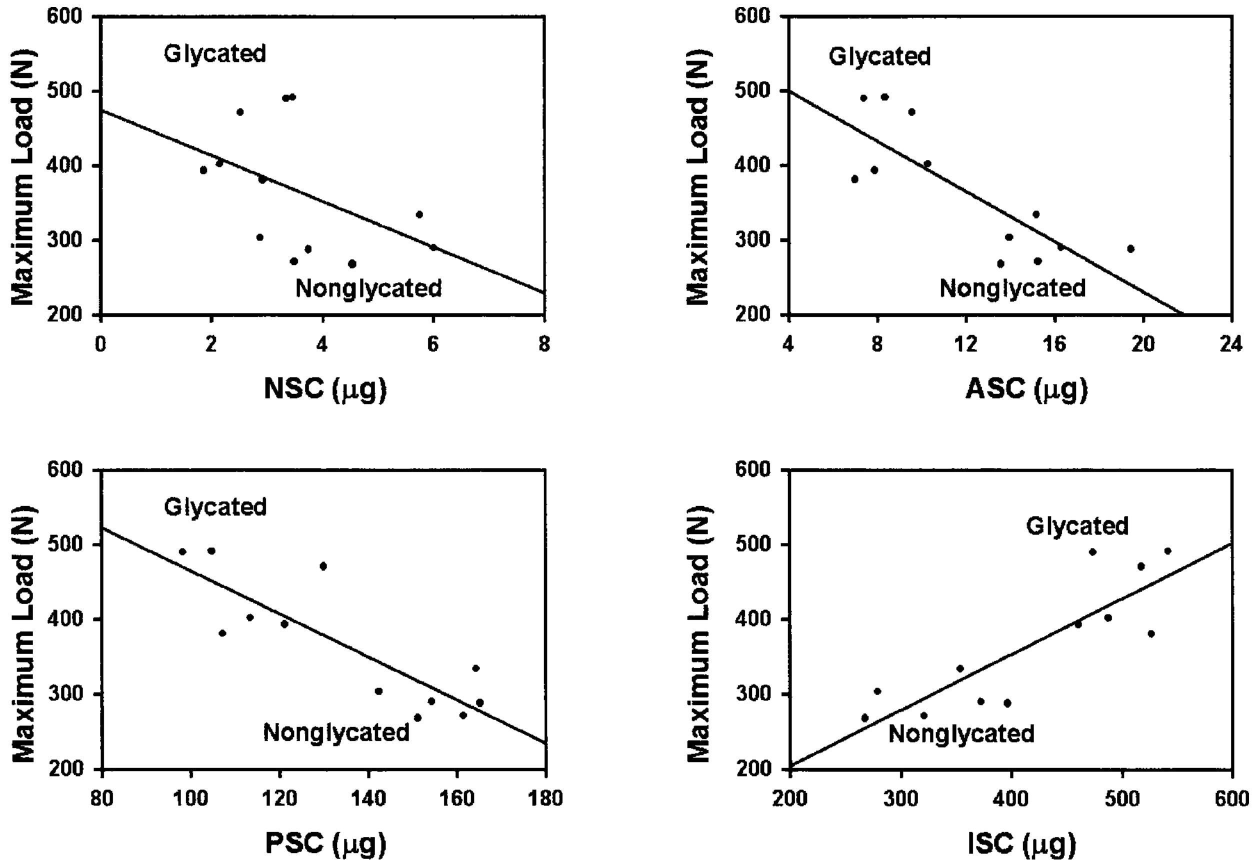

FIGURE 2
Scatterplots illustrating the correlation between maximum load and soluble and insoluble collagen contents from glycated and nonglycated tendons. Regression analysis showed that the maximum load of the tendons inversely correlated with the NSC ($r = .47$, $P < .01$), ASC ($r = .81$, $P < .01$), and PSC ($r = .84$, $P < .01$) contents. The ISC content of the tendon showed a direct correlation with the maximum load ($r = .85$, $P < .01$).

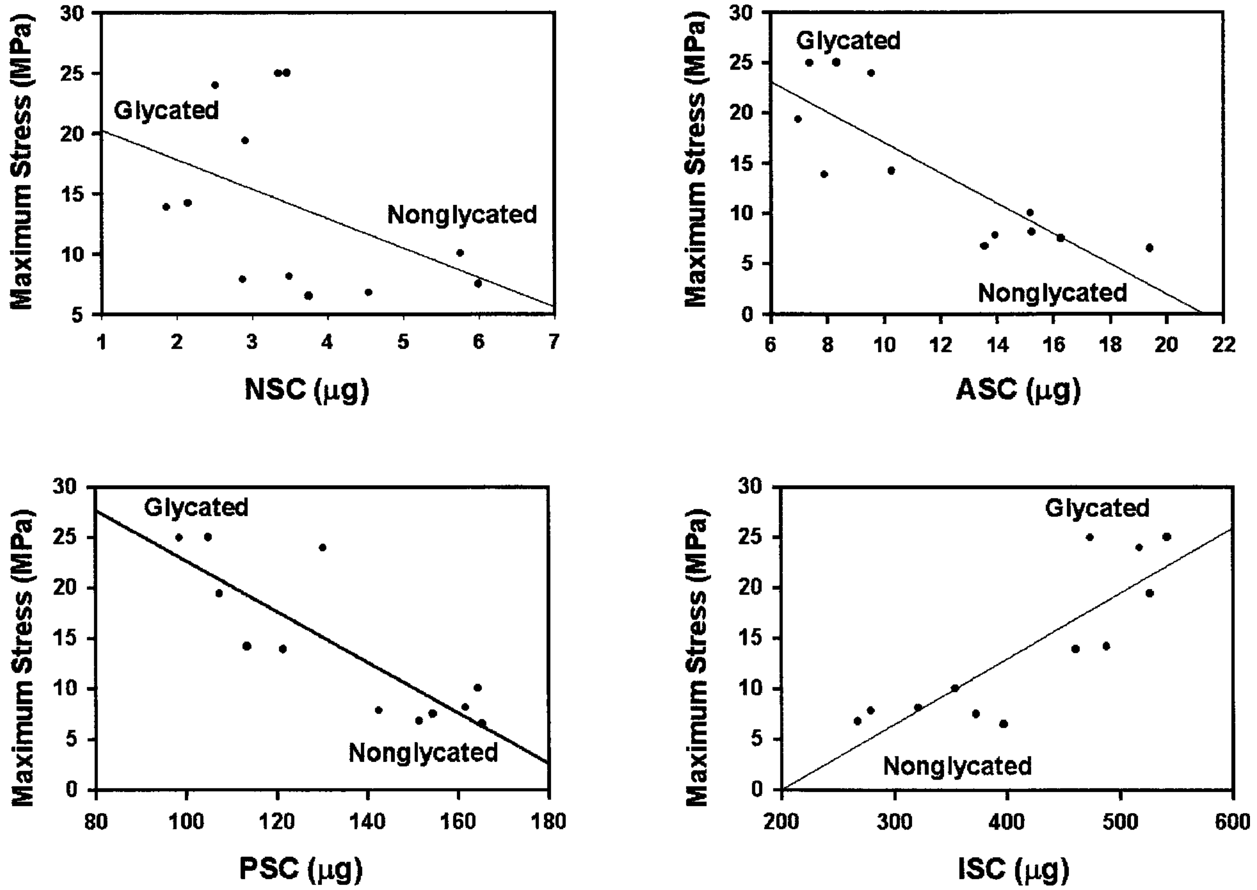

FIGURE 3
Scatterplots illustrating the correlation between the maximum stress and soluble and insoluble collagen contents from glycated and nonglycated tendons. Regression analysis showed that the maximum stress of the tendons inversely correlated with the NSC ($r = .43$, $P < .01$), ASC ($r = .83$, $P < .01$), and PSC ($r = .84$, $P < .01$) contents. The ISC content of the tendon showed a direct correlation with the maximum stress ($r = .85$, $P < .01$).

the biomechanical properties indicated that the maximum load withstood by the tendons was significantly higher in the glycated group compared to nonglycated group (50%, $P < .01$). Similarly, the maximum stress was significantly increased in glycated tendons compared to nonglycated tendons (114%, $P < .01$). However, the effect of glycation was found to be insignificant for maximum strain of the tendons (15%, $P > .05$). Young's modulus of elasticity for nonglycated tendons was significantly higher compared to glycated tendons (161%, $P < .01$).

The results in relation to energy absorption, i.e., energy to yield point, and toughness indicates that the absorption of energy in both elastic and plastic regions is significantly increased in glycated tendons compared to nonglycated tendons. This was evidenced by significant increases in energy to yield point (164%, $P < .01$) and toughness (176%, $P < .01$) in glycated tendons compared to nonglycated tendons.

The data presented above summarize the differences in the biomechanical integrity between glycated and nonglycated Achilles tendons at the maximum load point. The data collected at break point are summarized in Table 2. Significant differences were documented between the nonglycated and glycated tendons when measured for load at break and strain at break ($P < .05$). However, there were no statistically significant differences documented in other biomechanical characteristics, such as strain at break and energy absorption at break point, between the nonglycated and glycated Achilles tendons.

### Biochemical Measurements

The findings of the degree of collagen cross-linking, i.e., changes in NSC, ASC, PSC, ISC, and pentosidine contents, for the tendons are presented in Table 3. The findings showed that the solubility of the NSC from glycated tendons was reduced to 61% of the nonglycated tendons, suggesting that glycation-induced cross-links may be responsible for the reduction in NSC content ($P < .01$). Similarly, ASC and PSC of the tendons were

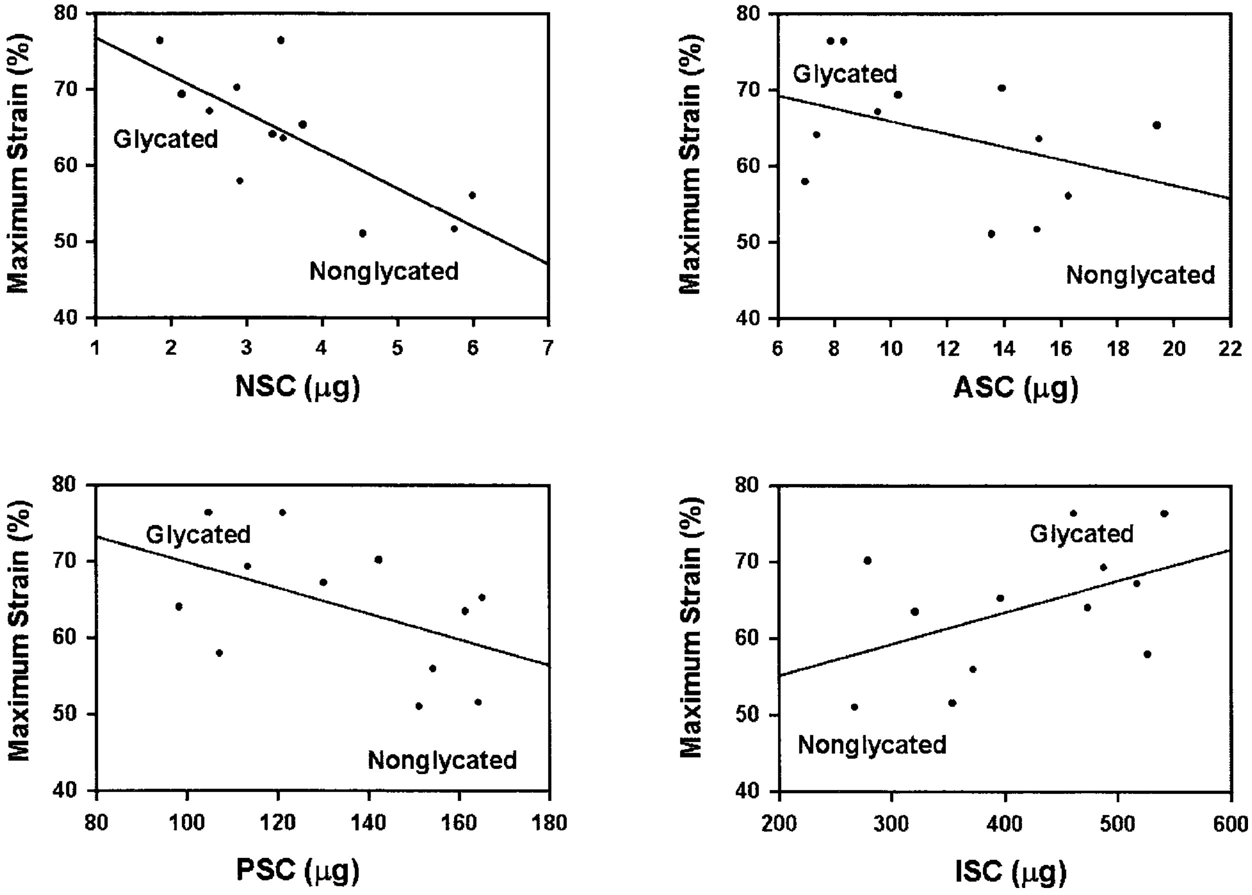

FIGURE 4
Scatterplots illustrating the correlation between the maximum strain and soluble and insoluble collagen contents from glycated and nonglycated tendons. Regression analysis showed that the maximum strain of the tendons inversely correlated with the NSC ($r = .75$, $P < .01$), ASC ($r = .40$, $P < .05$), and PSC ($r = .49$, $P < .01$) contents. The ISC content of the tendon showed a direct correlation with the maximum strain ($r = .79$, $P < .01$).

reduced to 48% and 28%, respectively, following glycation, indicating that the glycation process enhanced the resistance of tendons to acetate and pepsin treatments ($P < .01$). The amount of insoluble collagen was increased by 28% in glycated tendons compared to nonglycated tendons ($P < .01$). Furthermore, the formation of pentosidine was significantly increased in glycated tendons compared to nonglycated tendons (168%, $P < .01$).

The relative amounts of the distribution of soluble collagens in relation to total tissue collagen are shown in Figure 1. Approximately 0.5% and 0.8% of collagen was extractable in the neutral salt soluble fraction from glycated and nonglycated tendons, respectively. Using 0.5 M acetate buffer, 1.5% and 2.9% of collagen were extracted from glycated and nonglycated tendons, respectively. The results of pepsin-digested samples showed that a large amount of collagen was solubilized in both groups of tendons (20.5% for glycated tendons and 33.7% for nonglycated tendons). The percent of insoluble collagen was appreciably higher in glycated tendons (77.5%) than in nonglycated tendons (62.6%).

### Relationship Between Biomechanics and Biochemistry of Collagen

Figures 2 to 7 show the relationship between biomechanical and biochemical properties for glycated and nonglycated tendons. It is interesting to note that glycation-induced collagen cross-linking is strongly associated with the alterations of the matrix biomechanics in the tendon. The results clearly demonstrate that the maximum load of the glycated tendons exhibited a significant inverse correlation with the NSC ($r = .47$, $P < .01$), ASC ($r = .81$, $P < .01$), and PSC ($r = .84$, $P < .01$) contents of the tendons. Similarly, maximum stress of glycated tendons showed a negative relationship with the levels of NSC ($r = .43$, $P < .01$), ASC ($r = .83$, $P < .01$), and PSC ($r = .84$, $P < .01$). Maximum strain displayed

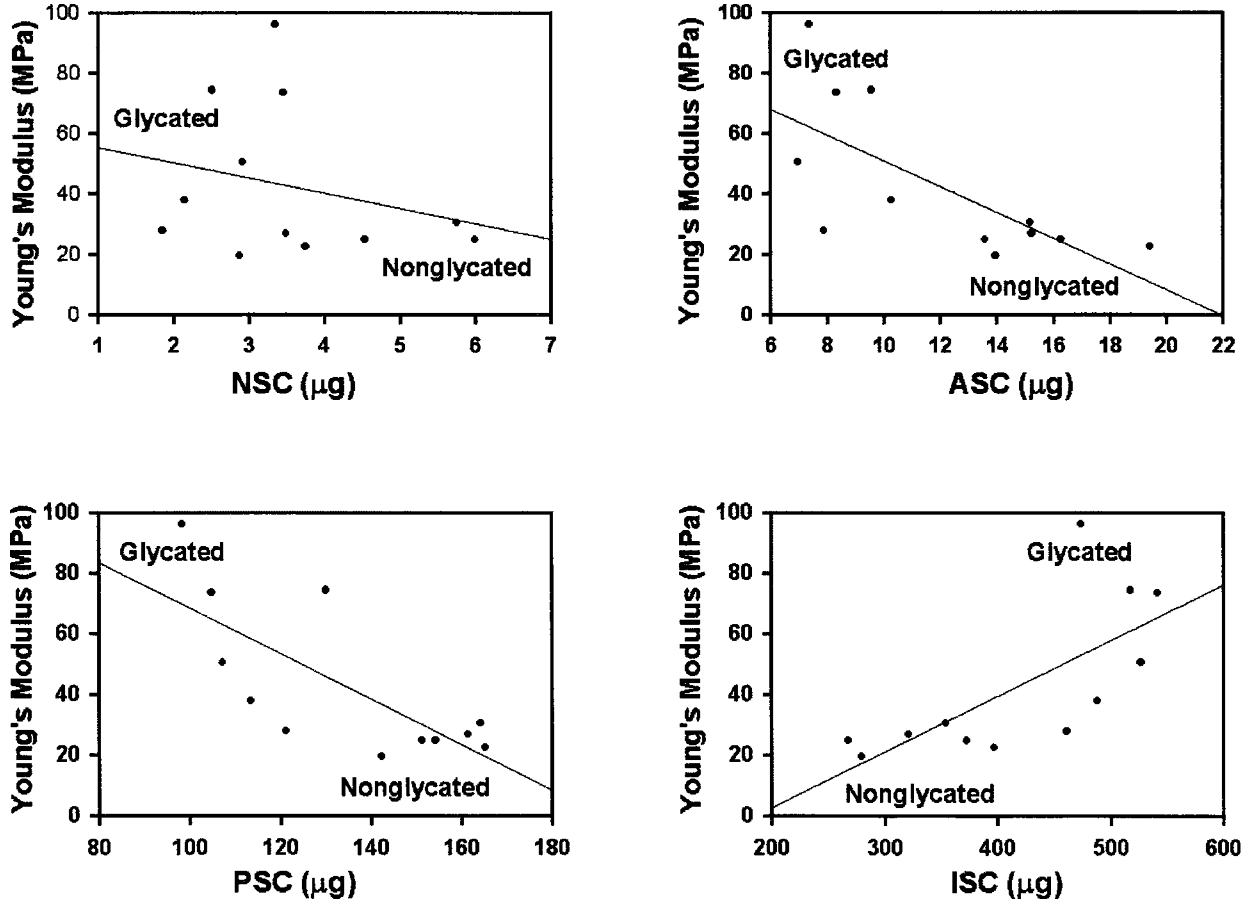

**FIGURE 5**
Scatterplots illustrating the correlation between the Young's modulus of elasticity and soluble and insoluble collagen contents from glycated and nonglycated tendons. Regression analysis showed that the Young's modulus of elasticity of the tendons inversely correlated with the NSC ($r = .22$, $P > .05$), ASC ($r = .65$, $P < .01$), and PSC ($r = .71$, $P < .01$) contents. The ISC content of the tendon showed a direct correlation with the Young's modulus of elasticity ($r = .61$, $P < .01$).

relatively a weak correlation with the ASC ($r = .40$, $P < .05$), PSC ($r = .49$, $P < .01$), and ISC ($r = .48$, $P > .05$) levels while exhibiting a strong inverse correlation with the NSC ($r = .75$, $P < .01$) content in glycated tendons. An inverse correlation was observed between the levels of NSC ($r = .22$, $P > .05$), ASC ($r = .65$, $P < .01$), and PSC ($r = .71$, $P < .01$) and Young's modulus of elasticity of the tendons. Similarly, the toughness of glycated tendons displayed a negative correlation with the NSC ($r = .49$, $P < .01$), ASC ($r = .77$, $P < .01$), and PSC ($r = .79$, $P < .01$) levels. However, the levels of insoluble collagen from glycated tendons showed a strong positive correlation with the maximum load (Figures 2 to 6; $r = .85$, $P < .01$), stress ($r = .85$, $P < .01$), strain ($r = .79$, $P < .01$), Young's modulus of elasticity ($r = .61$, $P < .01$), and toughness ($r = .89$, $P < .01$). The results presented in Figure 7 depict the relationship between the levels of pentosidine and various biomechanical attributes of the tendons. The pentosidine content correlated significantly with the maximum load ($r = .94$, $P < .01$), stress ($r = .92$, $P < .01$), and strain ($r = .76$, $P < .01$), Young's modulus of elasticity ($r = .75$, $P < .01$), toughness ($r = .94$, $P < .01$), and energy at break point ($r = .76$, $P < .01$). The impact of glycation showed a strong association between pentosidine and biomechanical attributes of the tendons.

### DISCUSSION

Nonenzymatic glycation of the tissue proteins and subsequent production of AGEs have been implicated in the process of normal aging as well as in the pathogenesis of several diseases, including diabetes [1, 3], atherosclerosis [8], fibromyalgia [9], uremia [10, 11], Alzheimer's disease [12, 13], and renal failure [14]. Compared to other tissues, connective tissue appears to be highly vulnerable to glycation in the body. Collagen, the main organic constituent of connective tissue matrix, and

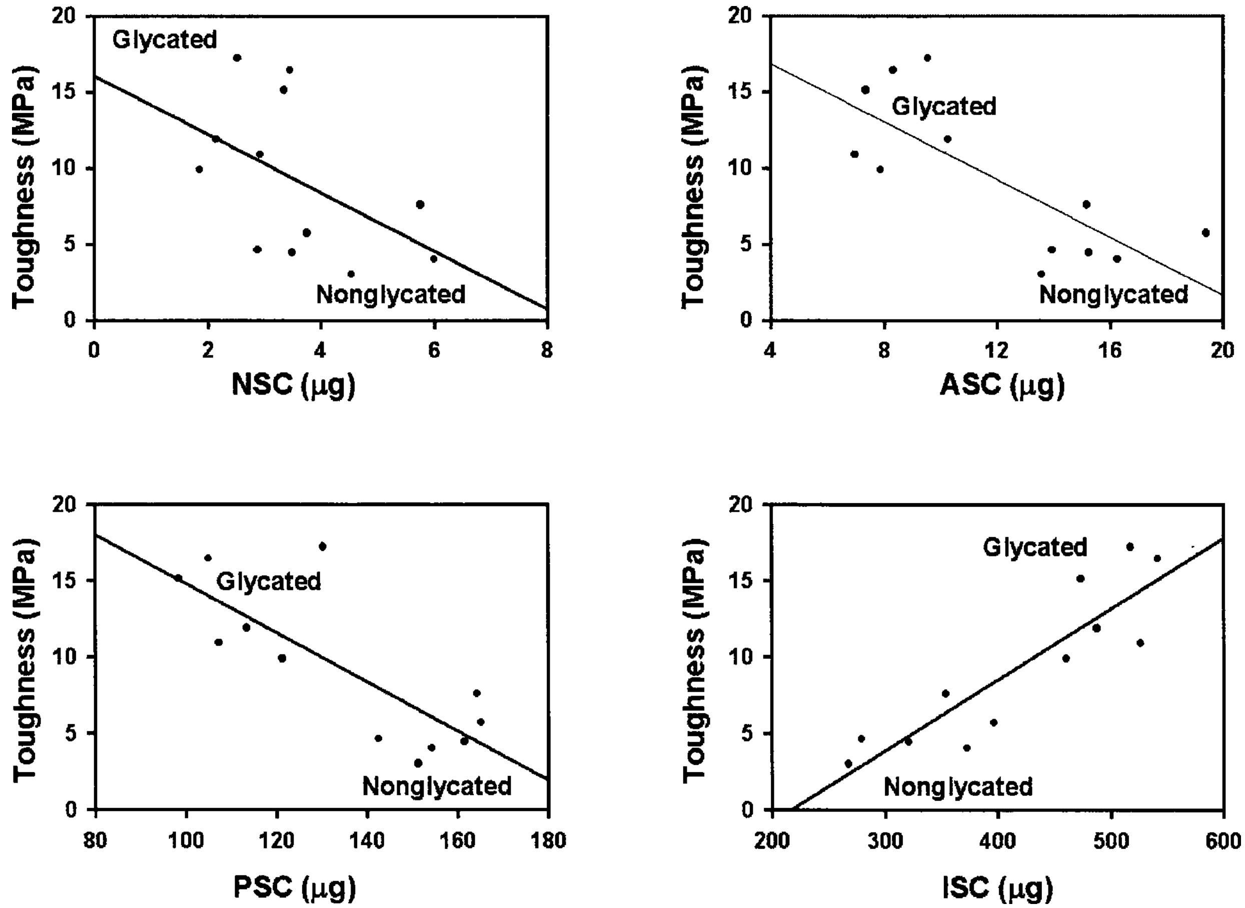

FIGURE 6

Scatterplots illustrating the correlation between the toughness and soluble and insoluble collagen contents from glycated and nonglycated tendons. Regression analysis showed that the toughness of the tendons inversely correlated with the NSC ($r = .49$, $P < .01$), ASC ($r = .77$, $P < .01$), and PSC ($r = .79$, $P < .01$) contents. The ISC content of the tendon showed a direct correlation with the toughness ($r = .89$, $P < .01$).

one of the major targets for nonenzymatic glycation, plays an important role in determining the functional properties of the tissue. Clinical complications associated with diabetes may result in functional impairment of the collagen-rich tissues, such as tendon, ligament, bone, skin, and the tunica adventitia of blood vessels, due to glycation. Recently, we have demonstrated that in vitro glycation increased the matrix stability of Achilles tendon. This study investigated not only the glycation-induced changes in the matrix stability of tissue but also the potential associations between the biomechanical attributes and biochemical properties of collagen in the tendon following an in vitro nonenzymatic glycation.

The biomechanical testing showed a significant increase in maximum load and stress, Young's modulus of elasticity, and toughness in glycated tendons compared to nonglycated tendons. The changes in the biomechanical properties of Achilles tendons by the process of nonenzymatic glycation are consistent with the previous findings reported by the author and colleagues [23] and other investigators [22, 29–31]. During biomechanical testing, the coiled or crimped collagen fibrils in the tendon are expected to align along the axis of loading. The tensile stress-strain response in tendon, in the physiological loading, is primarily due to the reorientation of bundles of fibers in the hydrated matrix [32]. The biomechanical integrity of tendon, therefore, has commonly been associated with the supramolecular organization and physical properties of the collagen fiber network.

The supramolecular organization of collagen in tendons and other tissues is primarily dependent on the degree of cross-linking. Nonenzymatic glycation is known to influence the cross-linking of collagen, and thus affect the biomechanical integrity of the tissue. In order to examine the influence of glycation on collagen cross-linking, the solubility of collagen was measured following sequential treatments with neutral and

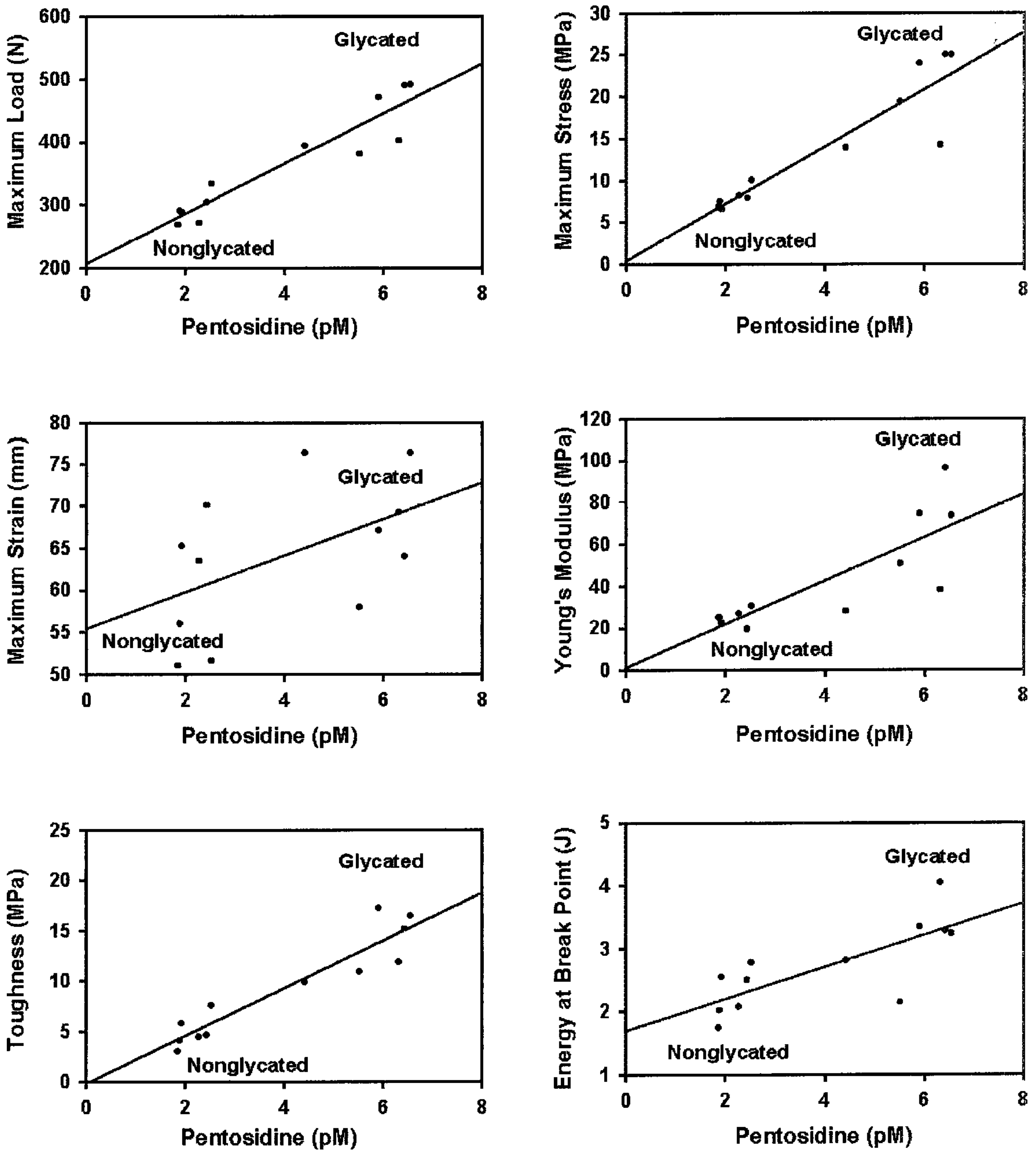

**FIGURE 7**

Scatterplots illustrating the correlation between the pentosidine and various biomechanical attributes of the glycated and nonglycated tendons. Regression analysis showed that the pentosidine content is directly associated with the maximum load ($r = .94$, $P < .01$), stress ($r = .92$, $P < .01$), strain ($r = .76$, $P < .01$), Young's modulus of elasticity ($r = .75$, $P < .01$), toughness ($r = .94$, $P < .01$), and energy at break point ($r = 0.76$, $P < .01$).

acetate buffer as well as with acetate buffer containing pepsin. The results of this study clearly demonstrate that the proportion of soluble collagen (in neutral buffer, weak acid, and pepsin digestible) was significantly lower in glycated tendon compared to nonglycated tendon.

The solubility of collagen in weak acid depends primarily on the extent of disruption of noncovalent and covalent bonds, but not stabilized aldimine cross-links [33]. Furthermore, collagen that is solubilized in weak acid contains more cross-links than salt-soluble collagen [34]. In this study, we observed a decrease in the proportions of acid-soluble collagen in glycated tendons compared to nonglycated tendons. Thus, the decrease in acid-soluble collagen content reflects the manifestations in acid-labile cross-links in collagen by the nonenzymatic process.

In addition, collagen in glycated tendons was highly resistive to pepsin digestion compared to nonglycated tendons. The nonhelical regions of the collagen molecules possess inter- and intramolecular cross-links [34, 35], and are the major sites for the cleavage by the proteolytic enzyme pepsin. Furthermore, the results of the collagen solubility assay demonstrate that insoluble collagen content of glycated tendons was increased significantly compared to nonglycated tendons, exhibiting marked differences in the susceptibility of collagen to proteolytic degradation.

It was our working hypothesis that the glycation-induced collagen modifications exhibit a high degree of correlation with the matrix stiffness of the tendons. This was found to be the case in that the degree of collagen cross-linking by nonenzymatic glycation showed a significant correlation with the biomechanical attributes of the tendons. The soluble collagens, such as NSC, ASC, and PSC, showed a significant inverse correlation with maximum load, stress, and strain, Young's modulus of elasticity, and toughness of the tendons. Insoluble collagen and pentosidine levels showed a strong positive correlation with the various biomechanical indices, including Young's modulus and toughness of the tendons. Young's modulus of elasticity, a direct marker of stiffness, showed a direct association with glycation cross-links, such as pentosidine, and insoluble collagen levels of the tendons. The toughness parameter that measures the energy absorption capacity of the tissue showed a direct correlation with the pentosidine and insoluble collagen levels of the tendons. Thus, the results of this study clearly demonstrate that the stiffness of the tissue directly correlated with insoluble collagen and pentosidine levels and indirectly correlated with NSC, ASC, and PSC levels of the glycated tendons.

There are several limitations to the present study. First, the results may be model or method specific. We used ribose for our in vitro study instead of glucose, which mimics to a greater extent the in vivo process of glycation during hyperglycemia and diabetes. Secondly, measurement of biomechanics revealed considerable variability in tissue stiffness. Moreover, the number of specimens was limited to 6 in each group. Due to the small sizes of the samples, such variability in the data causes difficulty with statistical analysis.

Based on our in vitro results, we conclude that glycation-induced cross-linking in collagen results in increased matrix stiffness as evaluated by measuring maximum load, stress, and strain, Young's modulus of elasticity, and toughness of the tendons. Although the levels of glycation-induced collagen cross-linking by in vivo process differs from this in vitro study, the accumulation of these collagen cross-links seems a plausible mechanism to explain the observed matrix stiffness in Achilles tendon.

### REFERENCES

[1] Monnier, V. M. (1989) Toward a Maillard reaction theory of aging. *Prog. Clin. Biol. Res.,* **304,** 1–22.

[2] Brownlee, M., Cerami, A., and Vlassara, H. (1988) Advanced glycosylation end products in tissue and the biochemical basis of diabetic complications. *N. Engl. J. Med.,* **318,** 1315–1321.

[3] Brownlee, M. (1995) Advanced protein glycosylation in diabetes and aging. *Annu. Rev. Med.,* **46,** 223–234.

[4] Brownlee, M., Vlassara, H., and Cerami, A. (1984) Nonenzymatic glycosylation and the pathogenesis of diabetic complications. *Ann. Intern. Med.,* **101,** 527–537.

[5] Schnider, S. L., and Kohn, R. R. (1981) Effects of age and diabetes mellitus on the solubility and nonenzymatic glucosylation of human skin collagen. *J. Clin. Invest.,* **67,** 1630–1635.

[6] Baynes, J. W., and Thorpe, S. R. (1999) Role of oxidative stress in diabetic complications: A new perspective on an old paradigm. *Diabetes.,* **48,** 1–9.

[7] Baynes, J. W. (2001) The role of AGEs in aging: Causation or correlation. *Exp. Gerontol.,* **36,** 1527–1537.

[8] Vlassara, H. (1996) Advanced glycation end-products and atherosclerosis. *Ann. Med.,* **28,** 419–426.

[9] Hein, G., and Franke, S. (2002) Are advanced glycation end-product-modified proteins of pathogenetic importance in fibromyalgia? *Rheumatology (Oxford),* **41,** 1163–1167.

[10] Schwedler, S., Schinzel, R., Vaith, P., and Wanner, C. (2001) Inflammation and advanced glycation end products in uremia: simple coexistence, potentiation or causal relationship? *Kidney Int. Suppl.,* **78,** S32–S36.

[11] Zoccali, C., Mallamaci, F., and Tripepi, G. (2000) AGEs and carbonyl stress: Potential pathogenetic factors of long-term uraemic complications. *Nephrol. Dial. Transplant.,* **15,** 7–11.

[12] Munch, G., Schinzel, R., Loske, C., Wong, A., Durany, N., Li, J. J., Vlassara, H., Smith, M. A., Perry, G., and Riederer, P. (1998) Alzheimer's disease—synergistic effects of glucose deficit, oxidative stress and advanced glycation endproducts. *J. Neural. Transm.,* **105,** 439–461.

[13] Colaco, C. A., Ledesma, M. D., Harrington, C. R., and Avila, J. (1996) The role of the Maillard reaction in other pathologies: Alzheimer's disease. *Nephrol. Dial. Transplant.,* **11,** 7–12.

[14] Vlassara, H. (1996) Protein glycation in the kidney: Role in diabetes and aging. *Kidney Int.,* **49,** 1795–1804.

[15] Brownlee, M. (1994) Lilly Lecture 1993. Glycation and diabetic complications. *Diabetes,* **43,** 836–841.

[16] Vlassara, H., Bucala, R., and Striker, L. (1994) Pathogenic effects of advanced glycosylation: biochemical, biologic, and clinical implications for diabetes and aging. *Lab. Invest.,* **70,** 138–151.

[17] Greenwald, D. P., Shumway, S., Zachary, L. S., LaBarbera, M., Albear, P., Temaner, M., and Gottlieb, L. J. (1993) Endogenous versus toxin-induced diabetes in rats: A mechanical comparison of two skin wound-healing models. *Plast. Reconstr. Surg.,* **91,** 1087–1093.

[18] Einhorn, T. A., Boskey, A. L., Gundberg, C. M., Vigorita, V. J., Devlin, V. J., and Beyer, M. M. (1988) The mineral and mechanical properties of bone in chronic experimental diabetes. *J. Orthop. Res.,* **6,** 317–333.

[19] Menzel, E. J., and Reihsner, R. (1991) Alterations of biochemical and biomechanical properties of rat tail tendons caused by non-enzymatic glycation and their inhibition by dibasic amino acids arginine and lysine. *Diabetologia.,* **34,** 12–16.

[20] Reiser, K. M. (1998) Nonenzymatic glycation of collagen in aging and diabetes. *Proc. Soc. Exp. Biol. Med.,* **218,** 23–37.

[21] Bellmunt, M. J., Portero, M., Pamplona. R., Cosso, L., Odetti, P., and Prat. J. (1995) Evidence for the Maillard reaction in rat lung collagen and its relationship with solubility and age. *Biochim. Biophys. Acta.,* **1272,** 53–60.

[22] Bai, P., Phua, K., Hardt, T., Cernadas, M., and Brodsky, B. (1992) Glycation alters collagen fibril organization. *Connect. Tissue. Res.,* **28,** 1–12.

[23] Reddy, G. K., Stehno-Bittel, L., and Enwemeka, C. S. (2002) Glycation-induced matrix stability in the rabbit Achilles tendon. *Arch. Biochem. Biophys.,* **399,** 174–180.

[24] Reddy, G. K., Stehno-Bittel, L., and Enwemeka, C. S. (1999) Matrix remodeling in healing rabbit Achilles tendon. *Wound. Repair. Regen.,* **7,** 518–527.

[25] Reddy, G. K., Stehno-Bittel, L., and Enwemeka, C. S. (1998) Laser photostimulation of collagen production in healing rabbit Achilles tendons. *Lasers. Surg. Med.,* **22,** 281–287.

[26] Reddy, G. K., Gum, S., Stehno-Bittel, L., and Enwemeka, C. S. (1998) Biochemistry and biomechanics of healing tendon: Part II. Effects of combined laser therapy and electrical stimulation. *Med. Sci. Sports. Exerc.,* **30,** 794–800.

[27] Reddy, G. K., and Enwemeka, C. S. (1996) A simplified method for the analysis of hydroxyproline in biological tissues. *Clin. Biochem.,* **29,** 225–229.

[28] Sell, D. R., and Monnier, V. M. (1989) Structure elucidation of a senescence cross-link from human extracellular matrix. Implication of pentoses in the aging process. *J. Biol. Chem.,* **264,** 21597–21602.

[29] Andreassen, T. T., Oxlund, H., and Danielsen, C. C. (1988) The influence of non-enzymatic glycosylation and formation of fluorescent reaction products on the mechanical properties of rat tail tendons. *Connect. Tissue. Res.,* **17,** 1–9.

[30] Andreassen, T. T., Seyer-Hansen, K., and Bailey, A. J. (1981) Thermal stability, mechanical properties and reducible cross-links of rat tail tendon in experimental diabetes. *Biochim. Biophys. Acta.,* **677,** 313–317.

[31] Galeski, A., Kastelic, J., Baer, E., and Kohn, R. .R (1977) Mechanical and structural changes in rat tail tendon induced by alloxan diabetes and aging. *J. Biomech.,* **10,** 775–782.

[32] Minns, R. J., Soden, P. D., and Jackson, D. S. (1973) The role of the fibrous components and ground substance in the mechanical properties of biological tissues: A preliminary investigation. *J. Biomech.,* **6,** 153–165.

[33] Weiss, J. B. (1976) Enzymic degradation of collagen. *Int. Rev. Connect. Tissue. Res.,* **7,** 101–157.

[34] Bornstein, P., and Traub, W. (1979) The chemistry and biology of collagen. In: *The Proteins,* Edited by Neurath, H., and Hill, R. L.), Vol. 4, pp. 411–632. New York, Academic Press.

[35] Schnider, S. L., and Kohn, R. R. (1982) Effects of age and diabetes mellitus on the solubility of collagen from human skin, tracheal cartilage and dura mater. *Exp. Gerontol.,* **17,** 185–194.

